# Epigenetic quantification of circulating immune cells in peripheral blood of triple-negative breast cancer patients

**DOI:** 10.1186/s13148-021-01196-1

**Published:** 2021-11-17

**Authors:** Mehdi Manoochehri, Thomas Hielscher, Nasim Borhani, Clarissa Gerhäuser, Olivia Fletcher, Anthony J. Swerdlow, Yon-Dschun Ko, Hiltrud Brauch, Thomas Brüning, Ute Hamann

**Affiliations:** 1 Molecular Genetics of Breast Cancer, German Cancer Research Center (DKFZ), Im Neuenheimer Feld 580, 69120 Heidelberg, Germany; 2 Department of in-Vitro Diagnostics, Fraunhofer Institute for Interfacial Engineering and Biotechnology IGB, Stuttgart, Germany; 3 Division of Biostatistics, German Cancer Research Center (DKFZ), Im Neuenheimer Feld 280, 69120 Heidelberg, Germany; 4 Cancer Epigenomics, German Cancer Research Center (DKFZ), Heidelberg, Germany; 5 The Breast Cancer Now Toby Robins Research Centre, The Institute of Cancer Research, London, UK; 6 The Institute of Cancer Research, London, UK; 7 Division of Genetics and Epidemiology and Division of Breast Cancer Research, The Institute of Cancer Research, London, UK; 8 Department of Internal Medicine, Evangelische Kliniken Bonn gGmbH, Johanniter Krankenhaus, 53113 Bonn, Germany; 9 Dr. Margarete Fischer-Bosch Institute of Clinical Pharmacology, 70376 Stuttgart, Germany; 10 iFIT Cluster of Excellence, University of Tübingen, 72074 Tübingen, Germany; 11 German Cancer Consortium (DKTK) and German Cancer Research Center (DKFZ), Partner Site Tübingen, 72074 Tübingen, Germany; 12 Institute for Prevention and Occupational Medicine of the German Social Accident Insurance, Institute of the Ruhr University Bochum (IPA), 44789 Bochum, Germany

**Keywords:** Triple-negative breast cancer, DNA methylation in blood, Immune cell subtypes, TNBC risk

## Abstract

**Background:**

A shift in the proportions of blood immune cells is a hallmark of cancer development. Here, we investigated whether methylation-derived immune cell type ratios and methylation-derived neutrophil-to-lymphocyte ratios (mdNLRs) are associated with triple-negative breast cancer (TNBC).

**Methods:**

Leukocyte subtype-specific unmethylated/methylated CpG sites were selected, and methylation levels at these sites were used as proxies for immune cell type proportions and mdNLR estimation in 231 TNBC cases and 231 age-matched controls. Data were validated using the Houseman deconvolution method. Additionally, the natural killer (NK) cell ratio was measured in a prospective sample set of 146 TNBC cases and 146 age-matched controls.

**Results:**

The mdNLRs were higher in TNBC cases compared with controls and associated with TNBC (odds ratio (OR) range (2.66–4.29), all *P*_adj._ < 1e−04). A higher neutrophil ratio and lower ratios of NK cells, CD4 + T cells, CD8 + T cells, monocytes, and B cells were associated with TNBC. The strongest association was observed with decreased NK cell ratio (OR range (1.28–1.42), all *P*_adj._ < 1e−04). The NK cell ratio was also significantly lower in pre-diagnostic samples of TNBC cases compared with controls (*P* = 0.019).

**Conclusion:**

This immunomethylomic study shows that a shift in the ratios/proportions of leukocyte subtypes is associated with TNBC, with decreased NK cell showing the strongest association. These findings improve our knowledge of the role of the immune system in TNBC and point to the possibility of using NK cell level as a non-invasive molecular marker for TNBC risk assessment, early detection, and prevention.

**Supplementary Information:**

The online version contains supplementary material available at 10.1186/s13148-021-01196-1.

## Introduction

Inflammation plays an important role in almost every stage of cancer development. Many inflammatory markers have been associated with cancer progression and prognosis [1]. Various studies showed that the number and function of blood leukocytes are altered in cancer [2–5]. A shift in the number of peripheral immune cells is a predictor of cancer patient survival. For instance, an increased neutrophil-to-lymphocyte ratio (NLR) which is indicative of systemic inflammation, could promote cancer cell proliferation, angiogenesis, cellular migration, and metastasis [6]. There is evidence from many studies for a prognostic role of NLR in peripheral blood of various cancer patients [7–10], including breast cancer [8, 11, 12].

Triple-negative breast cancer (TNBC) accounts for 15–20% of all breast cancers [13]. Due to the lack of targeted therapies, chemotherapy still is the main therapeutic strategy. Therefore, many efforts have been conducted to increase the diagnostic and therapeutic opportunities for the TNBC patients [14]. TNBC is also the most immunogenic subtype [15, 16]. Higher levels of infiltrated T cells are associated with an improved OS and disease-free survival (DFS) of TNBC patients as compared with those affected by other breast cancer subtypes [15, 16]. In another recent study, four spatially distinct tumor immune microenvironment subtypes defined by distinct CD8 + T cell localization patterns and gene expression signatures were identified, which were associated with distinct disease outcomes [17]. Further, NLR is associated with survival in a pre-treatment setting and throughout the treatment course and subsequent follow-up [5, 18, 19]. In addition, higher peripheral lymphocyte counts are associated with a lower mortality from early-stage TNBC suggesting that immune cell functions improve early TNBC treatment [20].

Epigenetic modifications such as DNA methylation play an important role in the cell-specific gene regulation within the hematopoietic system [21, 22]. Since DNA methylation signatures are chemically stable and mitotically heritable, they have been successfully applied to quantify leukocyte subtypes accurately in DNA from peripheral blood [23–25].

**Fig. 1:**
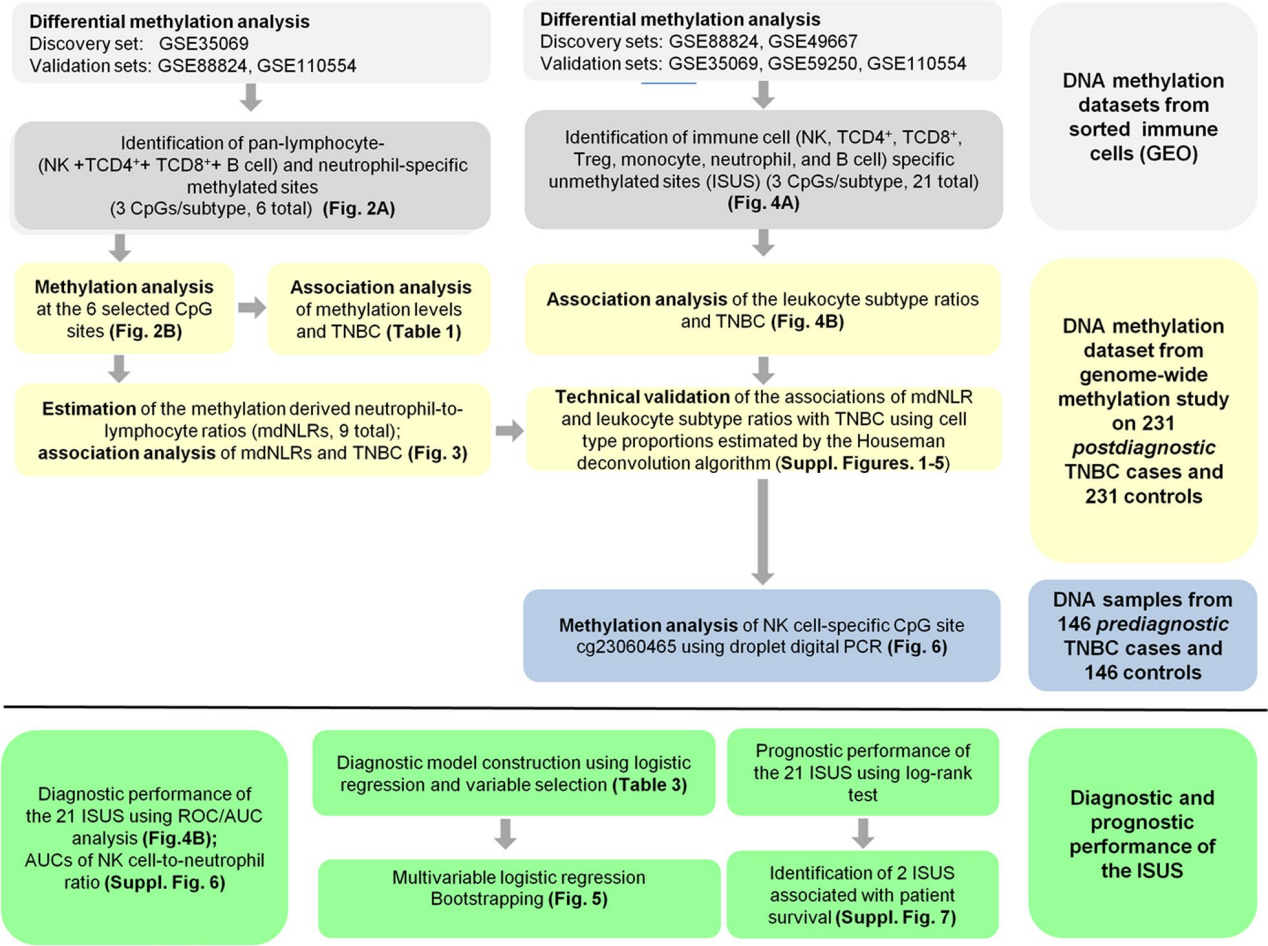
Graphical representation of the analyses performed in the study

In the present study, we identified and validated associations of methylation-derived leukocyte subtype ratios and methylation-derived neutrophil-to-lymphocyte ratios (mdNLRs) with TNBC using methylation data of 231 TNBC cases and 231 age-matched controls from a retrospective study. We report associations of various leukocyte subtypes ratios with TNBC, with the natural killer (NK) cell ratio showing the strongest association with disease. Further, we provide evidence for an association of the NK cell ratio with TNBC risk in a prospective sample set of 146 TNBC cases and 146 age-matched controls (for a graphical overview of the work, see Fig. 1).

## Methods

### Study populations

In the present study, two TNBC case–control sample sets were analyzed: One set from a retrospective study contains 231 TNBC cases and 231 age-matched controls selected from two different studies: the breast cancer case-only study SKKDKFZS [26] and the breast cancer case–control study GENICA [27]. The prospective sample set includes 146 TNBC cases and 146 controls from the Generations Study (GS) [28]. All study participants were women of European ethnicity. All studies had local ethical approval and all included individuals gave informed written consent. Details on the study populations can be found in Additional file: 9 (available online). The sample sizes and selected characteristics of study participants are presented in Additional file 1, 2: Tables S1 and S2.

### DNA methylation analysis

We applied publically available DNA methylation datasets obtained from isolated leukocyte subtypes (NK cells, CD4 + T cells (TCD4 +), CD8 + T cells (TCD8 +), regulatory T cells (Treg), monocytes, neutrophils, and B cells) for selection of cell type-specific differentially methylated sites. Thereafter, DNA methylation levels (beta values) at selected CpG sites were used as proxies for leukocyte subtypes proportions and mdNLRs. The mdNLR was estimated by dividing the beta value at neutrophil-specific CpGs by the beta value of pan-lymphocyte-specific CpGs. Details on the experimental strategies and data analysis are described in Additional file 9 (available online).

In-house genome-wide and locus-specific DNA methylation analysis was performed on the retrospective and prospective sample sets, respectively. Genome-wide DNA methylation profiling was performed on 231 TNBC cases and 231 controls from a retrospective study using the Illumina Infinium HumanMethylation450K BeadChip according to the manufacturer´s instructions. Subsequently, for measurement of the NK cell level in the prospective cohort of 146 TNBC cases and 146 controls, MethyLight droplet digital PCR (ddPCR) assay was carried out on bisulfite converted DNA. More details on the materials and methods are described in Additional file 9.

For technical validation of our findings, we applied a reference-based method (Houseman algorithm) to statistically deconvolute the proportions of six immune cell subtypes (NK, TCD4 + , TCD8 + cells, monocytes, neutrophils, and B cells) in the TNBC cases and controls [29]. The mdNLR_reference-based method_ (mdNLR_ref_) was calculated by dividing the estimated proportion of neutrophils by the sum of the lymphoid cell proportions (NK, TCD4 + , TCD8 + , and B cells).

### Statistical analyses

Associations of TNBC and leukocyte subtype ratios were analyzed using the methylation beta values from the retrospective study. The ratios of the seven leukocyte subtypes in TNBC cases and controls were calculated and compared using methylation levels (beta value) of 21 selected immune cell-specific unmethylated sites (ISUS). A higher level of methylation at each ISUS corresponds to lower ratio of the corresponding leukocyte subtype.

In primary analysis, the diagnostic performance of individual CpGs and subtype ratios on TNBC status was assessed with univariable conditional logistic regression accounting for age-matched pairs (age matching ± 1 year) and receiver operating characteristic (ROC)/area under the curve (AUC) analysis. In supportive analysis, adjusted effects were estimated based on a multivariable conditional logistic regression model additionally accounting for body mass index (BMI) (continuous), menopausal status (pre/peri, post), ever parous (yes, no), and smoking status (current) (yes, no). Complete data for 221 matched pairs were available for the supportive analysis.

A multivariable logistic regression model based on all pre-selected CpGs was fitted with backward selection at a significance level of 20% for staying in the model. Internal validation of the AUC for the multivariable model including variable selection was done using bootstrapping with 200 repetitions. To assess the diagnostic performance of subtype ratios, differences in logit-transformed beta values were analyzed. In TNBC cases, the association of methylation levels with clinical and epidemiological parameters (age, menopausal status, BMI, smoking status, ever parous and number of children), histopathological tumor parameters (grade, size, node status, stage) and overall survival (OS) was assessed and in controls associations with age, menopausal status, BMI, and smoking status. OS was defined as the time between TNBC diagnosis and death or last follow-up, whichever occurred first. Mann–Whitney test, Jonckheere–Terpstra trend test, and Spearman’s correlation coefficient were used to assess associations between methylation levels and clinical, epidemiological, and histopathological parameters. The impact of beta values on OS was analyzed in a Cox regression model. To account for established prognostic factors, a multivariable Cox regression model including age, tumor grade (G1/G2 vs G3), stage (0–4), tumor size (T1, T2, T3, T4) and N status (N0 vs N1) was fitted. Kaplan–Meier estimates and log-rank test were derived for methylation levels at median cutoff. Individual *P* values were adjusted for multiple testing using Holm correction to control the family-wise error rate. The obtained “unmethylation “ values from ddPCR experiment were logit-transformed and univariable conditional logistic regression accounting for age-matched and follow-up time matched pairs (age matching ± 5 years) as well as Wilcoxon signed-rank test were used to compare the ratios between cases and controls. All analyses have been done using R 3.6 with add-on packages rms, survival and pROC.

## Results

### Associations of mdNLRs with TNBC

**Fig. 2:**
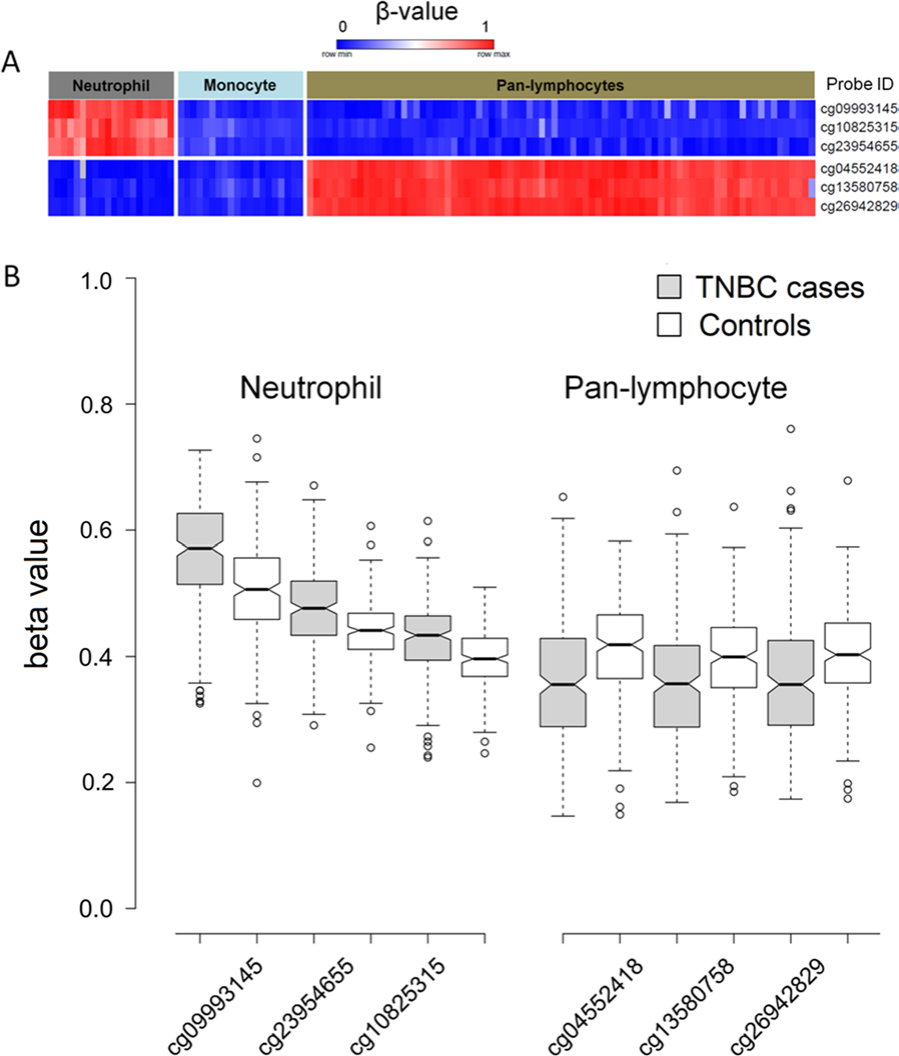
Heat map visualization of DNA methylation levels at the three pan-lymphocyte- and the three neutrophil-specific CpG sites using GEO datasets (**A**). Box plots of data on the methylation levels of the pan-lymphocyte- and neutrophil-specific CpG sites in 231 TNBC cases and 231 controls from the retrospective study (**B**). Outliers appear outside of the whiskers

The methylation levels of the selected neutrophil- and pan-lymphocyte-specific CpG sites are shown in Fig. 2A. The neutrophil-specific sites are methylated in the neutrophils and unmethylated in pan-lymphocytes (NK, TCD4 + , TCD8 + , and B cells) and monocytes. The pan-lymphocyte-specific sites are methylated in the pan-lymphocytes and unmethylated in neutrophils and monocytes. The characteristics of the CpG sites are provided in Table 1.

**Table 1:** Characteristics of selected CpG proxies in neutrophils and pan-lymphocytes

Cell type	Probe ID	Chromosome	Gene name	Genic region	*β*-value		OR [95% CI]^b^	*P*_adj_^c^	AUC [95% CI]
		Position^a^			Cases	Controls			
Neu	cg09993145	1:25291905	*RUNX3*	TSS1500	0.56	0.51	2.07 [1.60–2.67]	< 1e−04	0.68 [0.64–0.73]
	cg10825315	14:81425912	*TSHR*	Body	0.43	0.40	2.64 [1.80–3.87]	< 1e−04	0.67 [0.62–0.71]
	cg23954655	13:99223562	*STK24*	Body	0.48	0.44	3.02 [2.07–4.42]	< 1e−04	0.68 [0.63–0.73]
Pan-lym	cg04552418	1:31956405	Intergenic	–	0.36	0.41	0.56 [0.44–0.70]	< 1e−04	0.66 [0.62–0.71]
	cg13580758	4:57824450	*REST*	Body	0.36	0.40	0.58 [0.46–0.74]	< 1e−04	0.64 [0.59–0.69]
	cg26942829	6:13408158	*GFOD1*	Body	0.36	0.40	0.61 [0.49–0.77]	< 1e−04	0.65 [0.60–0.70]

OR, odds ratio; CI, confidence interval; *P*_adj_, adjusted *P* value; AUC, area under the curve; Neu, neutrophils; Pan-lym, pan-lymphocytes

^a^Human GRCh37/hg19 Assembly

^b^OR is given for a 10% increase in methylation level

^c^Adjusted for multiple testing using Holm correction

**Fig. 3:**
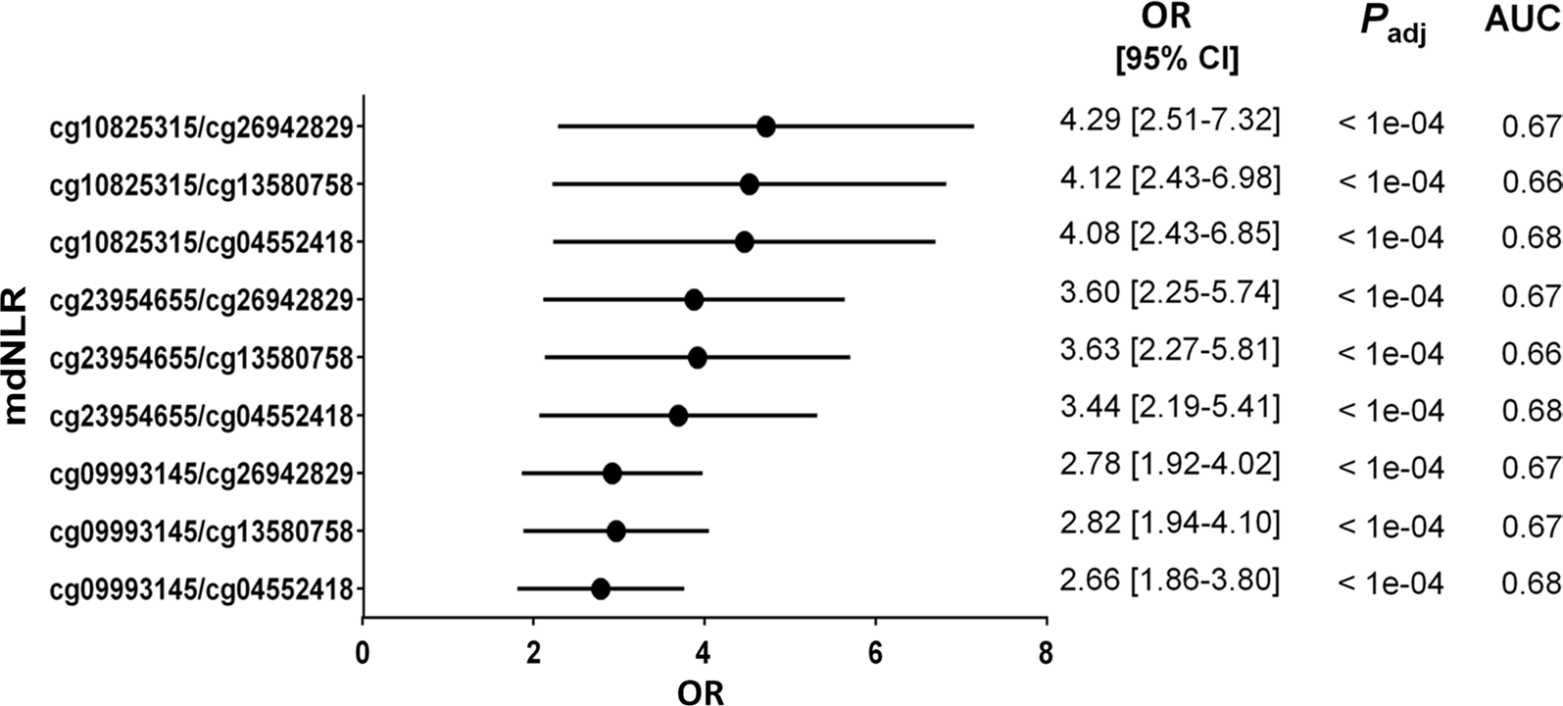
Forest plot of the associations of the nine mdNLRs with TNBC and estimated areas under the receiver operating characteristic curves (AUCs) with their corresponding 95% confidence intervals (CIs). OR is given for a 10% increase in methylation level. *P* values were adjusted (*P*_adj_) for multiple testing using the Holm correction. Horizontal lines indicate 95% CIs. mdNLR, methylation-derived neutrophil-to-lymphocyte ratio; OR, odds ratio

Methylation analysis at the selected CpG sites in 231 TNBC cases and 231 controls revealed a significantly higher ratio of neutrophils and lower ratio of pan-lymphocytes in TNBC cases compared with controls (mean ratios: 49% vs. 45% and 36% vs. 40.3%, respectively; conditional logistic regression: all *P*_adj._ < 1e−04*)* (Fig. 2B). The neutrophil ratios were associated with a higher likelihood of being a TNBC case (OR range (2.07–3.02); conditional logistic regression: all *P*_adj_. < 1e−04) and the pan-lymphocyte ratios with a lower likelihood (OR range (0.56–0.61); conditional logistic regression: all *P*_adj_. < 1e−04) (Additional file 3: Table S3). In cases, there were no associations of neutrophil and pan-lymphocyte ratios with epidemiological parameters, histopathological characteristics, and OS (size/stage: Jonckheere–Terpstra trend test; ever parous: Mann–Whitney test, number of children: Spearman’s correlation coefficient; Grade/Node status: Mann–Whitney test; OS: Cox regression; all *P* > 0.05). The average means of the mdNLR for cases and controls were 1.5 ± 0.63 and 1.18 ± 0.43, respectively. Logistic regression analysis revealed that all mdNLRs were associated with an increased likelihood of being a TNBC case (OR range (2.66–4.29); all *P*_adj._ < 1e−04) (Fig. 3). No associations were observed for any of the nine mdNLRs with epidemiological characteristics, histopathological characteristics, and OS.

To validate our mdNLR findings, we used the reference-based method (mdNLR_ref_), which estimates cell proportions. A higher mdNLR_ref_ was also observed in TNBC cases compared with controls (2.49 ± 1.53 versus 1.81 ± 0.94) (Additional file 8: Fig. S1), which was associated with a higher likelihood of being a TNBC case (OR = 1.71 [1.38–2.11], conditional logistic regression; *P*_adj._ < 1e−04) (Additional file 8: Fig. S2). The mdNLR and mdNLRs_ref_ were highly correlated (Additional file 8: Fig. S3), with the mdNLR cg23954655.cg26942829 ratio showing the highest correlation (Spearman’s rank correlation; r = 0.97).

Adjusting additionally for confounding factors (BMI, menopausal status, ever parous, smoking status) in a multivariable conditional logistic regression model, the associations of neutrophil and pan-lymphocyte ratios, mdNLRs, and mdNLR_ref_ with TNBC did not change fundamentally (Additional file 3–5: Tables S3–S5).

### Associations of leukocyte subtype ratios with TNBC

**Fig. 4:**
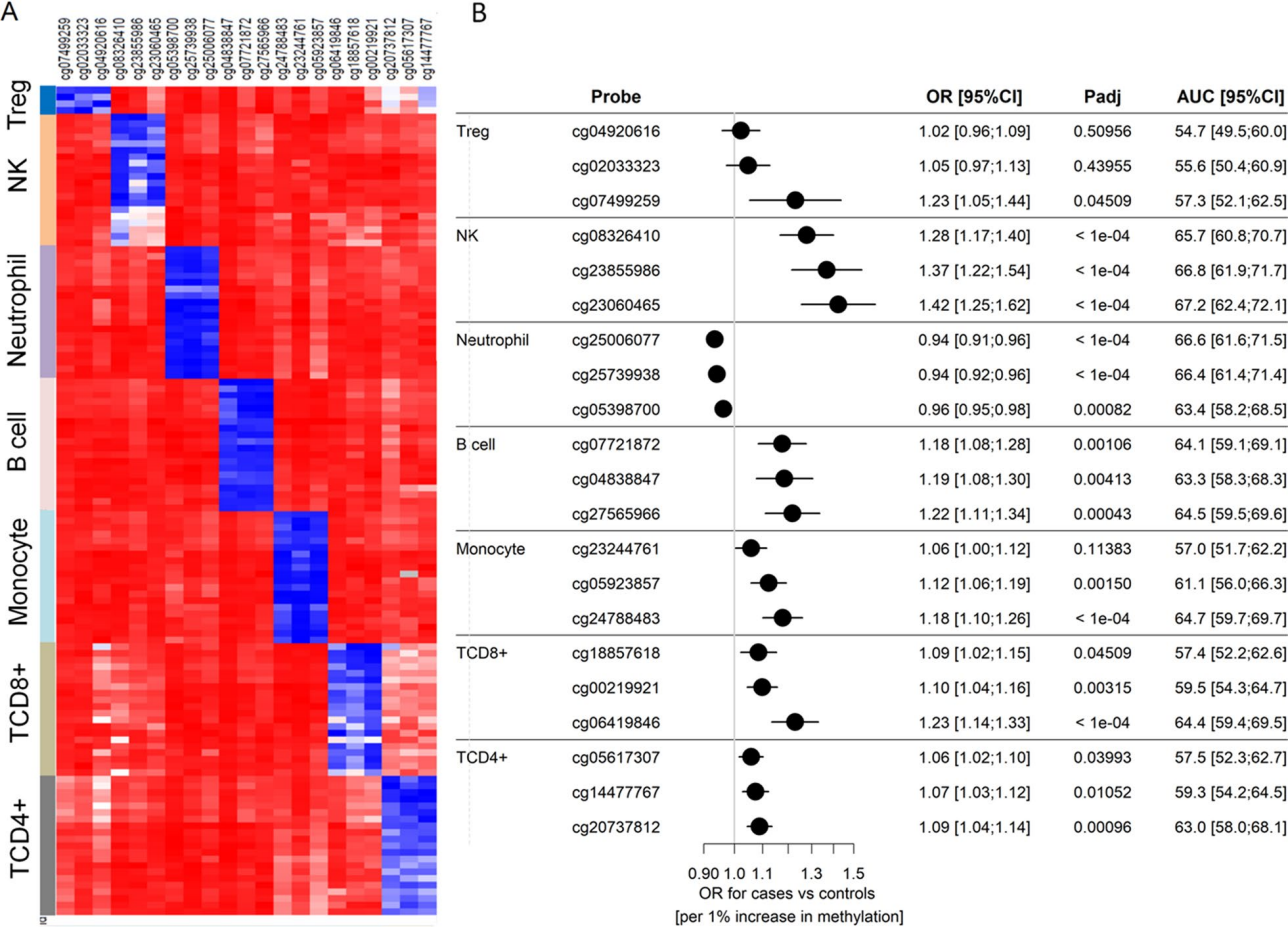
Heat map illustrating the methylation levels at the 21 selected ISUS in target immune cells versus other immune cells (**A**). Forest plot showing the associations of the leukocyte subtype ratios and TNBC using the ISUS methylation data and estimated areas under the receiver operating characteristic curves (AUCs) with their corresponding 95% confidence intervals (CIs) (**B**). *P* values were adjusted (*P*_adj_) for multiple testing using the Holm correction. Horizontal lines indicate 95% CIs. ISUS, immune cell-specific unmethylated site; OR, odds ratio

The methylation levels of the 21 selected ISUS are shown in Fig. 4A. All 21 ISUS were hypomethylated in the target cell types but methylated in the other subtypes. The characteristics of each ISUS are provided in Table 2. In order to investigate the association between leukocyte subtypes ratios with TNBC in the retrospective sample set, the immune cell type ratios of TNBC cases were compared with those in controls using beta values of the specific ISUS. Analysis of these proxies showed that six of seven immune cell type ratios were associated with TNBC. Lower ratios of NK, TCD4 + , TCD8 + cells, monocytes, and B cells in cases compared with controls were associated with TNBC (Fig. 4B), with decreased NK cell ratios showing the strongest association. Further, a higher ratio of neutrophils in TNBC cases compared with controls was associated with TNBC, while no difference between the two groups was found in the Treg cell ratio.

**Table 2:** Characteristics of selected ISUS as proxies and their participation in the diagnostic models

Cell type	ISUS Probe ID	Chromosome: Position^a^	Gene name	Genic region	LogReg	LogReg-VarSel
B cell	cg07721872	16:87735256	*LOC100129637*	Body	Yes	–
	cg04838847	8:110587155	*GOLSYN*	Body	Yes	–
	cg27565966	16:28943198	*CD19*	TSS200	Yes	–
Monocyte	cg23244761	6:161796850	*PARK2*	Body	Yes	–
	cg05923857	10:114911615	*TCF7L2*	Body	Yes	–
	cg24788483	10:114911652	*TCF7L2*	Body	Yes	Yes
TCD4 +	cg05617307	10:121413182	*BAG3*	Body	Yes	–
	cg14477767	4:170195438	Intergenic	–	Yes	–
	cg20737812	15:86336631	*KLHL25*	5'UTR	Yes	–
TCD8 +	cg18857618	2:87048489	*CD8B*	Body	Yes	–
	cg00219921	2:87012810	*CD8A*	3'UTR	Yes	–
	cg06419846	11:66083697	*CD248*	1stExon	Yes	Yes
Neu	cg25006077	3:152176018	*MBNL1*	Body	Yes	–
	cg25739938	2:9528072	*CPSF3*	Body	Yes	–
	cg05398700	14:102677141	*WDR20*	Body	Yes	–
NK	cg08326410	19:55314884	*KIR2DL4*	TSS200	Yes	Yes
	cg23855986	11:129980502	*APLP2*	Body	Yes	Yes
	cg23060465	8:141625545	*EIF2C2*	Body	Yes	–
Treg	cg04920616	X:49121288	*FOXP3*	TSS200	Yes	–
	cg02033323	X:49005257	*FOXP3*	5'UTR	Yes	–
	cg07499259	1:12188502	*TNFRSF8*	Body	Yes	–

Neu, neutrophil; NK, natural killer cell; Treg, regulatory T cell; LogReg, logistic regression; LogReg-VarSel, variable selection in logistic regression

^a^Human GRCh37/hg19) Assembly

To validate our findings obtained with the immune cell methylation proxies, we applied immune cell proportions estimated by reference-based deconvolution method. For each sample, the estimated proportions of the six immune cell types sum to one. Univariable comparison of the various immune cell types between TNBC cases and controls revealed a statistically significant difference in the proportions of neutrophils, NK, TCD4 + , and B cells (Additional file 8: Fig. S4). No difference was observed in the proportions of TCD8 + cells and monocytes. Logistic regression analysis showed associations of neutrophils, NK, TCD4 + , and B cell proportions with TNBC, with a decreased NK cell proportion showing the strongest association signal (Additional file 8: Fig. S2).

After adjustment for confounding factors, the associations of the leukocyte subtype ratios and proportions remained statistically significant except the association with cg07499259, which was no longer statistically significant (Additional file 5, 6: Tables S5 and S6).

### Correlation of leukocyte subtype ratios with clinical, epidemiological, and histopathological parameters of the participants of the retrospective study

Correlation analysis of the immune cell type ratios with selected clinical and epidemiological characteristics of the study participants revealed correlations with smoking status and age. The neutrophil and B cell ratios correlated with smoking status in controls. Current smokers had a lower neutrophil and a higher B cell ratio compared with non-smokers (Mann–Whitney test; *P*_adj_ < 0.05). Two immune cell type ratios correlated with age. The TCD8 + cell ratio showed an inverse correlation with age in both cases and controls and the NK cell ratio a positive correlation in controls (Spearman’s rank correlation; *P*_adj_ < 0.05). No other correlations were observed. In TNBC cases, there were also no correlations of the immune cell type ratios with ever parous, number of children, and histopathological tumor characteristics (grade, size, node status, stage).

### Diagnostic and prognostic performance of ISUS

AUC analysis showed that NK cells and neutrophils had the highest discriminative capability among all immune cell types (Fig. 4B). The estimated NK cell-to-neutrophil ratio was higher in controls compared with cases and slightly improved the discrimination performance between cases and controls with AUC values in the range (0.67–0.71) (Additional file 8: Fig. S5) relative to the values from individual CpGs in the range (0.63–0.67) (Fig. 4B). The NK cell-to-neutrophil ratios were associated with TNBC: a higher NK cell-to-neutrophil ratio was associated with a lower likelihood of being a TNBC cases (OR range (0.52-0-70); conditional logistic regression; all *P*_adj_. < 1e−04) (Additional file 7: Table S7). Next, a diagnostic model was developed by fitting a multivariable logistic regression model based on all 21 ISUS and applying backward variable selection. A bootstrap-adjusted AUC was computed to account for overfitting. The final model contained four ISUS that discriminated cases from controls with an AUC of 72%. Of these four ISUS, two were specific for NK cells, one for monocytes, and one for TCD8 + cells (Table 3).

**Table 3:** Multivariable logistic regression after variable selection

CpG ID	Cell type	OR [95% CI]	*P*
cg08326410	NK	1.14 [1.04–1.26]	0.00592
cg23855986	NK	1.31 [1.15–1.49]	< 1e−04
cg24788483	Monocyte	1.17 [1.09–1.25]	< 1e−04
cg06419846	TCD8 +	1.17 [1.09–1.26]	< 1e−04

OR, odds ratio; CI, confidence interval; NK, natural killer cell

The prognostic performance using log-rank test showed that two probes, cg00219921 and cg08326410, which are specific for TCD8 + and NK cells, were associated with survival when using a median split. Higher ratios of TCD8 + and NK cells were associated with a better patient OS (Additional file 8: Fig. S6). Using a multivariable Cox regression model including age, tumor grade, stage, tumor size, and lymph node status, only the association with cg00219921 remained statistically significant (Cox regression; *P* = 0.04). However, after adjustment for multiple testing, the association lost statistical significance.

### Association of the NK cell ratio with TNBC in participants of the prospective case–control study

**Fig. 5:**
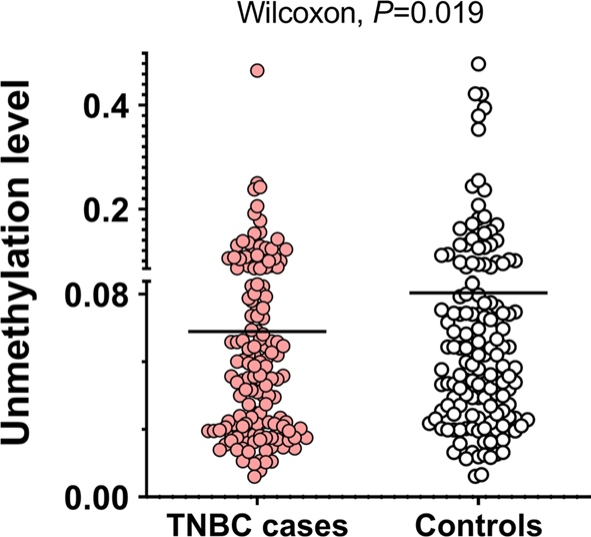
Scatter plot of the DNA “unmethylation” ratio in prediagnosed TNBC cases and controls. The unmethylation level corresponds to the NK cell ratio in sample groups

Since NK cells were the most pronounced immune cell type associated with TNBC in the retrospective sample set, we investigated whether the observed association could be detected in pre-diagnostic DNA samples of TNBC cases compared with controls. In this respect, the NK cell ratio was measured in 146 TNBC cases and 146 controls using a ddPCR TaqMan assay specific for one NK cell-specific unmethylated site (cg23060465). The obtained “unmethylation” levels from ddPCR were Logit-transformed and the Wilcoxon signed-rank test was used to compare the obtained NK cell ratios between cases and controls. A lower NK cell ratio was observed in TNBC cases compared with controls (Wilcoxon signed-rank test, *P* = 0.019) (Fig. 5). Using conditional logistic regression, a higher NK cell ratio was associated with a reduced TNBC risk at the margin of statistical significance (OR = 0.76, 95% CI [0.58–1.00], conditional logistic regression; *P* = 0.052). Heterogeneity due to age at blood draw, age of diagnosis, and interval time between blood draw and reference date was tested. No heterogeneity/subgroup effect was observed (Interaction test based on conditional logistic regression; all *P* > 0.05).

## Discussion

In the present large immunomethylomic study, we showed that methylation-derived leukocyte subtype ratios and mdNLRs at subtype-specific CpG sites are associated with TNBC in peripheral blood of TNBC patients and age-matched controls from a retrospective study. We showed that mdNLRs were higher in TNBC cases compared with controls and associated with TNBC. Further, higher ratios of neutrophils and lower ratios of NK, TCD4 + , TCD8 + , monocytes, and B cells were associated with TNBC, with a decreased NK cell ratio showing the strongest association. Associations of mdNLRs and neutrophil, NK, TCD4 + , and B cell ratios were validated in analysis based on immune cell type proportions. Moreover, we confirmed that the NK cell ratio was significantly lower in pre-diagnostic samples of TNBC cases compared with controls.

Overall, a 4% higher ratio of neutrophils and a 4.3% lower ratio of total lymphocytes were observed in TNBC cases compared with controls. It was reported that breast tumors maximize their chance of metastasizing by evoking a systemic inflammatory cascade, which leads to an elevated level of neutrophils. These tumor-induced neutrophils suppress cytotoxic TCD8 + lymphocytes, which ultimately enhancing metastatic seeding in the pre-metastatic lung [30]. Neutrophils also infiltrate many other tumor types, and the tumor microenvironment controls neutrophil recruitment [31]. They may act as potent antitumor effector cells (N1 neutrophils) via direct cytotoxicity or by the activation of different innate and adaptive immune cells. Alternatively, neutrophils also may acquire a protumor activity (N2 neutrophils). This is now supported by a growing number of studies showing that neutrophils correlate with poor cancer prognosis [32]. Unfortunately, due to the lack of N1- and N2-specific methylation datasets of neutrophils, the estimation of these subtypes of neutrophils was not feasible in our sample cohort.

Furthermore, the interactions between neutrophils and lymphocytes play critical roles in carcinogenesis. As a hallmark of cancer, in this study also an elevated mdNLR was observed in cases compared with controls (1.5 vs. 1.18). With slight difference, this result was repeated also by Houseman estimation method (averaged mdNLR_ref_ for cases and controls were 2.49 and 1.81, respectively). This finding agrees with that from a previous study, which reported mdNLR values of 2.7 in TNBC cases and 2.4 in controls [3]. A high NLR was associated with adverse survival of patients affected by various solid tumors, including TNBC [5, 7, 9, 18]. However, in contrast to the previous studies, mdNLR was not an independent predictor of OS in our study. The discrepant results obtained in these studies may be explained by differences in the study size, study population, and the methods used for mdNLR estimation.

Among the major immune cell types, higher neutrophil and lower ratios of B cells, TCD4 + , TCD8 + , NK cells, and monocytes were associated with TNBC. NK cell ratio showed the strongest association in the large retrospective sample set of 231 TNBC cases and 231 controls. Since the blood samples were drawn after the diagnosis of TNBC, it cannot be excluded that the shift in the NK cell ratio was induced by the tumor. There is evidence from a previous study that the tumor itself could manipulate and decrease the numbers of immune cells, such as NK cells, in the peripheral blood of TNBC patients by secreting cytokines [33]. However, a lower NK cell ratio was also observed in the prospective sample set of 146 TNBC cases compared with controls, which was associated with a higher TNBC risk at the margin of statistical significance. This finding suggests that the shift in NK cell ratio has occurred before disease manifestation. Interestingly, a lower NK cell level was also reported in head and neck cancer patients compared with controls with individuals in the lowest NK tertile having over fivefold risk of being a case [2].

There is evidence that NK cell levels are linked with survival of cancer patients. One study demonstrated that the NK cell ratio in the peripheral blood is an independent predictor of survival of colorectal cancer patients; those with a higher percentage of NK cells being associated with a better survival than those with a lower percentage [10]. Higher peripheral NK cell counts were also associated with better OS in lymphoma and chronic lymphocytic leukemia patients [34–36]. Further, there is some evidence that the number of NK cells in peripheral blood may affect the outcome of B cell non-Hodgkin lymphoma patients receiving immunochemotherapy [37].

NK cells are effector lymphocytes of the innate immune system that control several types of tumors and microbial infections by limiting their spread [38]. They have a crucial role in the control of metastasis by virtue of killing circulating tumor cells and acting as the first line of defense against metastasis from circulating tumor cells [39]. Therefore, the lower numbers of circulating NK cells in patients with TNBC may be an underlying cause of the disease, and individuals with decreased NK cell levels could be predisposed to develop TNBC. One previous study reported that mice with deficiencies in NK cell number and function are more susceptible to transplanted tumors [40]. In addition, it has been shown before that individuals with low cytotoxic activity of peripheral blood lymphocytes, including NK cells, are at higher risk of developing various types of cancer [41].

Impairment of NK cell function was also reported to play a role in breast tumorigenesis [42]. Breast tumors modify their environment to evade NK cell antitumor immunity [43] and ex vivo-expanded NK cells showed potent antitumor function against breast cancer cell lines and primary cells isolated from patients [44, 45]. Another study suggested that NK cells are important players in TNBC development and metastasis that could be used as a promising immunotherapeutic against TNBC [46]. In this respect, increasing NK cell antitumor activity and ex vivo expanding NK cell populations is paving the way for a new generation of anticancer immunotherapies [4]. Our findings may highlight the value of NK cell-based immunotherapies for TNBC; given that NK cell-induced lysis was significantly higher in TNBC cell lines compared to estrogen receptor positive breast cancer cell lines [47]. In addition to NK and T cells, peripheral blood also contains NKT cells, which are considered at the interface between innate and adaptive immunity. In contrast to NK cells, which are CD3-negative and CD56-positive, NKT cells express both CD3 and CD56 and were independently associated with cancer survival [48]. To the best of our knowledge, the genome-wide methylation profiling of NKT cells is not available at the present time. Given the lack of NKT cell-specific DNA methylation markers to be used as proxy for this immune cell type, an estimation of the NKT cells in our sample set was not possible. At present, flow cytometry is the most widely applied analytical approach for immune cell quantification [25]. This method, however, is limited to intact cells, but fresh or well-preserved blood samples are not available for many clinical cohorts in which samples were often collected in numerous centers, different geographic regions, and within a time period of several years. Therefore, in various recent studies, including this one, an epigenetic assay has been used [2, 3, 23, 25], which allows cell quantification in samples of limited quality and quantity, is applicable to archival blood or DNA samples, which are available for many clinical cohorts, and is less costly than flow cytometry. On the other hand, applying genome-wide DNA methylation analysis to estimate the immune cell ratios by data deconvolution demands high costs and expertise, which may not be available for some clinical studies. It has been shown that the epigenetic assay performs equivalently to flow cytometry for immune cell quantification [23–25].

As is the case for all studies, this work is not without some limitations. One limitation is that epigenetic data could not be validated using classical cell counting by flow cytometry due to the lack of fresh blood samples from TNBC cases and controls. Another limitation is the still relatively limited set of only main immune cell subtypes that were investigated. Another limitation is that although the developed diagnostic model with four selected methylation markers improved the capability to distinguish patients with TNBC from controls to an AUC of 72% (compared with AUC values of individual CpGs in the range of 55% to 67%), this has no clinical utility based on a threshold AUC of > 80%. A higher discriminative capability has to be achieved by integrating other molecular markers of different sources such as cell-free nucleic acids or proteins in future models of non-invasive diagnosis of TNBC. Additionally, single cell expression and DNA methylation profiling of peripheral immune cells, especially NK cells, in TNBC patients and healthy controls may reveal unique NK cell states or signatures in individuals developing TNBC.

## Conclusion

In summary, this is the largest study investigating immune cell profiles in TNBC patients and controls using methylation data. We identified and validated associations of mdNLRs and neutrophil, TCD4 + , B, and NK cell subtype ratios/proportions with TNBC, with the latter having the strongest association signal. The NK cell ratio was also significantly lower in pre-diagnostic samples of TNBC cases compared with controls. Future studies on the peripheral NK cell population in TNBC patients may provide NK cell-based cellular/molecular signatures that may be useful as a potential non-invasive blood-based biomarker for TNBC risk assessment, early detection or immunotherapeutic applications.

## Supplementary Information

**Additional file 1: Table S1**. Selected characteristics of the TNBC cases and controls from the retrospective study and breast tumor parameters

**Additional file 2: Table S2**. Selected characteristics of the TNBC cases and controls from the prospective cohort

**Additional file 3: Table S3**. Associations of the neutrophil and pan-lymphocyte ratios with TNBC after adjustment for multiple testing and confounders

**Additional file 4: Table S4**. Associations of the nine mdNLRs with TNBC after adjustment for confounders

**Additional file 5: Table S5**. Associations of the immune cell type proportions and mdNLRref with TNBC after adjustment for confounders

**Additional file 6: Table S6**. Associations of the leukocyte subtype ratios and TNBC after adjustment for confounders

**Additional file 7: Table S7**. Associations of NK cell-to-neutrophil ratios with TNBC after adjustment for multiple testing and confounders

**Additional file 8: Fig. S1**. Distribution of the mdNLRref between TNBC cases and controls using the reference-based Houseman method. **Fig. S2**. Forest plot of the associations of the immune cell subtype proportions and mdNLR_ref_ with TNBC. Odds ratio (OR) is given for a 10% increase in methylation level. *P* values were adjusted (*P*_adj_) for multiple testing using the Holm correction. Horizontal lines indicate 95% CIs.**Fig. S3**. Spearman's rank correlation scatter plot of mdNLR and mdNLR_ref_ levels in peripheral blood samples of TNBC cases and controls. **Fig. S4**. Violin plots showing leukocyte subtype proportions in TNBC cases and controls estimated by Houseman method. **Fig. S5**. Area under the ROC curve of NK cell-to-neutrophil ratio in TNBC cases versus controls. **Fig. S6**. Kaplan–Meier survival plots of cg08326419 and cg00219921 with prognostic value.

**Additional file 9**. Supplementary methods

## Data Availability

The datasets of sorted immune cells analyzed during the current study are available in the Gene Expression Omnibus repository (GEO; https://www.ncbi.nlm.nih.gov/geo/) through GEO accessions: GSE35969, GSE59250, GSE110554, GSE88824, and GSE49667. The in-house generated methylation array data are not publically available. They can be accessed for research purposes upon request.

## Abbreviations

TNBC: Triple-negative breast cancer
NK cell: Natural killer cell
mdNLR: Methylation-derived neutrophil-to-lymphocyte ratio
OR: Odds ratio
ISUS: Immune cell-specific unmethylated sites
ROC: Receiver operating characteristic
AUC: Area under the curve
OS: Overall survival
ddPCR: Droplet digital PCR
mdNLR_ref_: Methylation-derived neutrophil-to-lymphocyte ratio estimated by Housman method
