# Supplementary material for: Epigenetic quantification of circulating immune cells in peripheral blood of triple-negative breast cancer patients
Source: Clin Epigenetics. 2021 Nov 17;13:207. doi: 10.1186/s13148-021-01196-1 (PMC8596937; doi:10.1186/s13148-021-01196-1)
Supplement: Supplementary file 1 — Additional file 1: Table S1. Selected characteristics of the TNBC cases and controls from the retrospective study and breast tumor parameters [file 13148_2021_1196_MOESM1_ESM.docx]

**Supplementary Table 1.** Selected characteristics of the TNBC cases and controls from the retrospective study and breast tumor parameters

| Characteristics | Cases N=231 | Controls N=231 | |
| --- | --- | --- | --- |
|  | **N (%)** | | **N (%)** |
| *Clinical and epidemiological* |  | |  |
| Age (years) |  | |  |
| Mean (SD) | 57.4 (11.3) | | 57.4 (11.4) |
| Median (range) | 58.3 (30.2-72.7) | | 58 (30-80) |
| Body mass index (kg/m^2^) |  | |  |
| Median (range) | 25.6 (17.7-40) | | 24.1(16.8-43) |
| Smoking status (current) |  | |  |
| Yes | 41 (17.8) | | 55 (23.8) |
| No | 185 (80) | | 176 (76.2) |
| Unknown | 5 (2.2) | | - |
| Menopausal status |  | |  |
| Premenopausal | 63 (27.2) | | 57 (24.7) |
| Postmenopausal | 166 (71.9) | | 169 (73.1) |
| Unknown | 2 (0.9) | | 5 (2.2) |
| Ever parous |  | |  |
| Yes | 193 (83.5) | | 183 (79.2) |
| No | 32 (13.9) | | 48 (20.8) |
| Unknown | 6 (2.6) | | - |
| Number of children |  | |  |
| 0 | 32 (13.9) | | ~~-~~ |
| 1 | 49 (21.2) | | ~~-~~ |
| 2 | 96 (41.6) | | ~~-~~ |
| 3+ | 48 (20.8) | | ~~-~~ |
| Unknown | 6 (2.6) | | ~~-~~ |
| Age at birth of last child (years), N=199 |  | |  |
| <30 | 71 (35.7) | | ~~-~~ |
| 30<40 | 51 (25.6) | | ~~-~~ |
| 40<50 | 1 (0.5) | | ~~-~~ |
| Unknown | 76 (38.2) | | ~~-~~ |
| *Tumor parameters and follow-up* |  | |  |
| Histological grade |  | | - |
| G1 | 7 (3) | | - |
| G2 | 98 (42.6) | | - |
| G3 | 125 (54.4) | | - |
| Unknown | 1 (0.4) | | - |
| Tumor size, T |  | |  |
| Tis | 3 (1.3) | | - |
| T1 | 96 (42.1) | | - |
| T2 | 116 (50.8) | | - |
| T3 | 6 (2.6) | | - |
| T4 | 10 (4.3) | | - |
| Node status, N |  | |  |
| N0 | 126 (54.5) | | - |
| N1 | 99 (42.9) | | - |
| Unknown | 6 (2.6) | | - |
| Stage |  | |  |
| 0 | 3 (1.3) | | - |
| 1 | 53 (22.9) | | - |
| 2 | 116 (50.2) | | - |
| 3 | 37 (16) | | - |
| 4 | 11 (4.8) | | - |
| Unknown | 11 (4.8) | | - |
| Follow up (years) |  | |  |
| Mean (SD) | 6.5 (3.8) | | - |
| Median (range) | 6.5 (0-15.9) | | - |

SD: standard deviation.
