# Supplementary material for: Epigenetic quantification of circulating immune cells in peripheral blood of triple-negative breast cancer patients
Source: Clin Epigenetics. 2021 Nov 17;13:207. doi: 10.1186/s13148-021-01196-1 (PMC8596937; doi:10.1186/s13148-021-01196-1)
Supplement: Supplementary file 2 — Additional file 2: Table S2. Selected characteristics of the TNBC cases and controls from the prospective cohort [file 13148_2021_1196_MOESM2_ESM.docx]

**Supplementary Table 2.** Selected characteristics of the TNBC cases and controls from the prospective cohort

| **Characteristics** | **Cases N=146** | **Controls N=146** |
| --- | --- | --- |
|  | **N (%)** | **N (%)** |
| Year of study entry | 2004–2010 | 2004–2009 |
| Age at study entry |  |  |
| Mean (SD) | 51.5 (11.6) | 51.5 (11.7) |
| Median (range) | 53 (24–80) | 54 (22–81) |
| Age at blood draw (years) |  |  |
| Mean (SD) | 51.5 (11.6) | 51.6 (11.7) |
| Median (range) | 53 (24–80) | 54 (22–81) |
| Age at diagnosis (years) |  |  |
| Mean (SD) | 56.3 (11.8) | - |
| Median (range) | 57.5 (26-87) | - |
| Blood draw to diagnosis (years) |  |  |
| Mean (SD) | 4.8 (2.5) | - |
| Median (range) | 4.5 (0–9) | - |

SD: standard deviation.
