# Supplementary material for: Epigenetic quantification of circulating immune cells in peripheral blood of triple-negative breast cancer patients
Source: Clin Epigenetics. 2021 Nov 17;13:207. doi: 10.1186/s13148-021-01196-1 (PMC8596937; doi:10.1186/s13148-021-01196-1)
Supplement: Supplementary file 3 — Additional file 3: Table S3. Associations of the neutrophil and pan-lymphocyte ratios with TNBC after adjustment for multiple testing and confounders [file 13148_2021_1196_MOESM3_ESM.docx]

| **Supplementary Table 3.** Associations of the neutrophil and pan-lymphocyte ratios with TNBC after adjustment for multiple testing and confounders | | | | | | |
| --- | --- | --- | --- | --- | --- | --- |
|  |  |  |  |  |  |  |
| **CpG site** | **OR [95% CI]** | ***P*** | ***P_adj_*^a^** | **OR [95% CI]** | ***P*** | ***P_adj_*^b^** |
| cg04552418 | 0.56 [0.44-0.70] | < 1e-04 | < 1e-04 | 0.54 [0.42-0.68] | < 1e-04 | < 1e-04 |
| cg13580758 | 0.58 [0.46-0.74] | < 1e-04 | < 1e-04 | 0.57 [0.44-0.73] | < 1e-04 | < 1e-04 |
| cg26942829 | 0.61 [0.49-0.77] | < 1e-04 | < 1e-04 | 0.60 [0.47-0.76] | < 1e-04 | < 1e-04 |
| cg09993145 | 2.07 [1.60-2.67] | < 1e-04 | < 1e-04 | 2.10 [1.61-2.74] | < 1e-04 | < 1e-04 |
| cg23954655 | 3.02 [2.07-4.42] | < 1e-04 | < 1e-04 | 3.14 [2.09-4.70] | < 1e-04 | < 1e-04 |
| cg10825315 | 2.64 [1.80-3.87] | < 1e-04 | < 1e-04 | 2.57 [1.74-3.81] | < 1e-04 | < 1e-04 |
| ^a^Adjusted for multiple testing using Holm correction. | | | |  |  |  |
| ^b^Adjusted for body mass index, menopausal status, and smoking status (current). | | | | | |  |
