# Supplementary material for: Epigenetic quantification of circulating immune cells in peripheral blood of triple-negative breast cancer patients
Source: Clin Epigenetics. 2021 Nov 17;13:207. doi: 10.1186/s13148-021-01196-1 (PMC8596937; doi:10.1186/s13148-021-01196-1)
Supplement: Supplementary file 4 — Additional file 4: Table S4. Associations of the nine mdNLRs with TNBC after adjustment for confounders [file 13148_2021_1196_MOESM4_ESM.docx]

| **Supplementary Table 4**. Associations of the nine mdNLRs with TNBC after adjustment for confounders | | | |
| --- | --- | --- | --- |
| **mdNLR** | **OR [95% CI]** | ***P*** | ***P_adj_*^a^** |
| cg10825315.cg26942829.ratio | 4.29 [2.51-7.32] | < 1e-04 | < 1e-04 |
| cg10825315.cg13580758.ratio | 4.12 [2.43-6.98] | < 1e-04 | < 1e-04 |
| cg10825315.cg04552418.ratio | 4.08 [2.43-6.85] | < 1e-04 | < 1e-04 |
| cg23954655.cg13580758.ratio | 3.63 [2.27-5.81] | < 1e-04 | < 1e-04 |
| cg23954655.cg26942829.ratio | 3.60 [2.25-5.74] | < 1e-04 | < 1e-04 |
| cg23954655.cg04552418.ratio | 3.44 [2.19-5.41] | < 1e-04 | < 1e-04 |
| cg09993145.cg13580758.ratio | 2.82 [1.94-4.10] | < 1e-04 | < 1e-04 |
| cg09993145.cg26942829.ratio | 2.78 [1.92-4.02] | < 1e-04 | < 1e-04 |
| cg09993145.cg04552418.ratio | 2.66 [1.86-3.80] | < 1e-04 | < 1e-04 |
