# Supplementary material for: Epigenetic quantification of circulating immune cells in peripheral blood of triple-negative breast cancer patients
Source: Clin Epigenetics. 2021 Nov 17;13:207. doi: 10.1186/s13148-021-01196-1 (PMC8596937; doi:10.1186/s13148-021-01196-1)
Supplement: Supplementary file 5 — Additional file 5: Table S5. Associations of the immune cell type proportions and mdNLRref with TNBC after adjustment for confounders [file 13148_2021_1196_MOESM5_ESM.docx]

| **Supplementary Table 5.** Associations of the immune cell type proportions and mdNLR_ref_ with TNBC after adjustment for confounders | | | |  |
| --- | --- | --- | --- | --- |
|  |  |  |  |  |
| **Subtype** | **OR [95% CI]** | ***P*** | ***P_adj_*^a^** |  |
| Neutrophil | 1.79 [1.44-2.24] | < 1e-04 | < 1e-04 |  |
| Pan-lym | 0.57 [0.46-0.71] | < 1e-04 | < 1e-04 |  |
| NK | 0.12 [0.05-0.30] | < 1e-04 | < 1e-04 |  |
| TCD4+ | 0.67 [0.53-0.85] | 0.00092 | 0.00366 |  |
| B cell | 0.35 [0.17-0.72] | 0.00386 | 0.01159 |  |
| TCD8+ | 0.82 [0.59-1.14] | 0.24569 | 0.49137 |  |
| Monocyte | 1.01 [0.47-2.18] | 0.98484 | 0.98484 |  |
| mdNLR_ref_ | 1.71 [1.38-2.11] | < 1e-04 | < 1e-04 |  |
| ^a^Adjusted for body mass index, ever parous, menopausal status, and smoking status (current). | | | |  |
