# Supplementary material for: Epigenetic quantification of circulating immune cells in peripheral blood of triple-negative breast cancer patients
Source: Clin Epigenetics. 2021 Nov 17;13:207. doi: 10.1186/s13148-021-01196-1 (PMC8596937; doi:10.1186/s13148-021-01196-1)
Supplement: Supplementary file 6 — Additional file 6: Table S6. Associations of the leukocyte subtype ratios and TNBC after adjustment for confounders [file 13148_2021_1196_MOESM6_ESM.docx]

| **Supplementary Table 6**. Associations of the leukocyte subtype ratios and TNBC after adjustment for confounders | | | | | | | |
| --- | --- | --- | --- | --- | --- | --- | --- |
|  |  |  |  |  |  |  |  |
| **CpG site** | **OR [95% CI]** | ***P*** | ***P_adj_*^a^** |  |  |  |  |
| cg04920616 | 1.00 [0.94-1.07] | 0.91948 | 0.91948 |  |  |  |  |
| cg02033323 | 1.05 [0.97-1.13] | 0.19739 | 0.39478 |  |  |  |  |
| cg07499259 | 1.21 [1.03-1.42] | 0.02275 | 0.09099 |  |  |  |  |
| cg08326410 | 1.30 [1.18-1.43] | < 1e-04 | < 1e-04 |  |  |  |  |
| cg23855986 | 1.36 [1.20-1.54] | < 1e-04 | < 1e-04 |  |  |  |  |
| cg23060465 | 1.40 [1.23-1.60] | < 1e-04 | < 1e-04 |  |  |  |  |
| cg25006077 | 0.93 [0.91-0.96] | < 1e-04 | < 1e-04 |  |  |  |  |
| cg25739938 | 0.94 [0.92-0.96] | < 1e-04 | < 1e-04 |  |  |  |  |
| cg05398700 | 0.96 [0.94-0.98] | 0.00011 | 0.00137 |  |  |  |  |
| cg07721872 | 1.17 [1.08-1.28] | 0.00025 | 0.00258 |  |  |  |  |
| cg04838847 | 1.18 [1.07-1.31] | 0.00152 | 0.01066 |  |  |  |  |
| cg27565966 | 1.22 [1.11-1.35] | < 1e-04 | 0.00079 |  |  |  |  |
| cg23244761 | 1.05 [0.99-1.11] | 0.08276 | 0.24829 |  |  |  |  |
| cg05923857 | 1.12 [1.05-1.19] | 0.00039 | 0.00349 |  |  |  |  |
| cg24788483 | 1.17 [1.09-1.26] | < 1e-04 | 0.00018 |  |  |  |  |
| cg18857618 | 1.10 [1.03-1.17] | 0.00542 | 0.02708 |  |  |  |  |
| cg00219921 | 1.11 [1.05-1.17] | 0.00023 | 0.00258 |  |  |  |  |
| cg06419846 | 1.26 [1.15-1.37] | < 1e-04 | < 1e-04 |  |  |  |  |
| cg05617307 | 1.07 [1.02-1.11] | 0.00441 | 0.02647 |  |  |  |  |
| cg14477767 | 1.08 [1.03-1.14] | 0.00101 | 0.00806 |  |  |  |  |
| cg20737812 | 1.09 [1.04-1.14] | < 1e-04 | 0.00130 |  |  |  |  |
| ^a^Adjusted for body mass index, menopausal status, and smoking status (current). | | | | | |  |  |
