# Supplementary material for: Epigenetic quantification of circulating immune cells in peripheral blood of triple-negative breast cancer patients
Source: Clin Epigenetics. 2021 Nov 17;13:207. doi: 10.1186/s13148-021-01196-1 (PMC8596937; doi:10.1186/s13148-021-01196-1)
Supplement: Supplementary file 7 — Additional file 7: Table S7. Associations of NK cell-to-neutrophil ratios with TNBC after adjustment for multiple testing and confounders [file 13148_2021_1196_MOESM7_ESM.docx]

| **Supplementary Table 7.** Associations of NK cell-to-neutrophil ratios with TNBC after adjustment for multiple testing and confounders | | | | | | | | |
| --- | --- | --- | --- | --- | --- | --- | --- | --- |
|  |  |  |  |  |  |  |  |  |
| **NK cell-to-neutrophil ratios** | **OR [95% CI]** | ***P*** | ***P_adj_*^a^** | **OR [95% CI]** | ***P*** | ***P_adj_^b^*** |  |  |
| cg05398700.cg08326410.ratio | 0.70 [0.60-0.82] | < 1e-04 | < 1e-04 | 0.70 [0.59-0.82] | < 1e-04 | < 1e-04 |  |  |
| cg05398700.cg23855986.ratio | 0.70 [0.60-0.82] | < 1e-04 | < 1e-04 | 0.70 [0.59-0.83] | < 1e-04 | < 1e-04 |  |  |
| cg05398700.cg23060465.ratio | 0.69 [0.58-0.81] | < 1e-04 | < 1e-04 | 0.69 [0.57-0.82] | < 1e-04 | < 1e-04 |  |  |
| cg25739938.cg08326410.ratio | 0.58 [0.48-0.71] | < 1e-04 | < 1e-04 | 0.58 [0.47-0.71] | < 1e-04 | < 1e-04 |  |  |
| cg25739938.cg23855986.ratio | 0.58 [0.47-0.71] | < 1e-04 | < 1e-04 | 0.57 [0.46-0.71] | < 1e-04 | < 1e-04 |  |  |
| cg25739938.cg23060465.ratio | 0.56 [0.45-0.70] | < 1e-04 | < 1e-04 | 0.56 [0.44-0.70] | < 1e-04 | < 1e-04 |  |  |
| cg25006077.cg08326410.ratio | 0.55 [0.45-0.68] | < 1e-04 | < 1e-04 | 0.54 [0.44-0.68] | < 1e-04 | < 1e-04 |  |  |
| cg25006077.cg23855986.ratio | 0.54 [0.44-0.67] | < 1e-04 | < 1e-04 | 0.54 [0.43-0.67] | < 1e-04 | < 1e-04 |  |  |
| cg25006077.cg23060465.ratio | 0.52 [0.42-0.66] | < 1e-04 | < 1e-04 | 0.52 [0.41-0.66] | < 1e-04 | < 1e-04 |  |  |
| ^a^Adjusted for multiple testing using Holm correction. | |  |  |  |  |  |  |  |
| ^b^Adjusted for body mass index, menopausal status, and smoking status (current). | | | |  |  |  |  |  |
