# Supplementary material for: Epigenetic quantification of circulating immune cells in peripheral blood of triple-negative breast cancer patients
Source: Clin Epigenetics. 2021 Nov 17;13:207. doi: 10.1186/s13148-021-01196-1 (PMC8596937; doi:10.1186/s13148-021-01196-1)

**Supplementary Figure 1.** Distribution of the mdNLR_ref_ between TNBC cases and controls using the reference-based Houseman method.


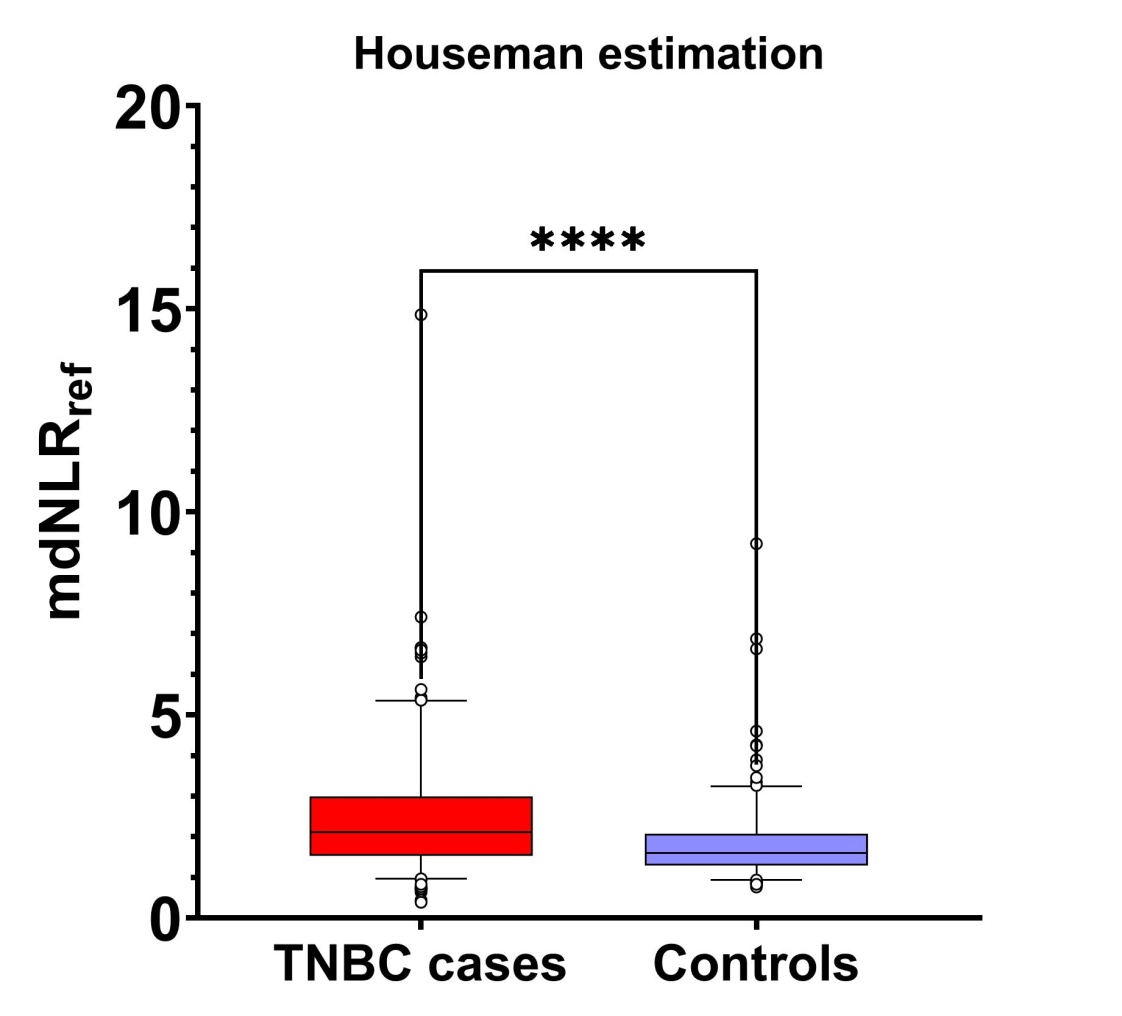


**Supplementary Figure 2.** Forest plot of the associations of the immune cell subtype proportions and mdNLR_ref_ with TNBC. Odds ratio (OR) is given for a 10% increase in methylation level. *P*-values were adjusted (*P_adj_*) for multiple testing using the Holm correction. Horizontal lines indicate 95% CIs.


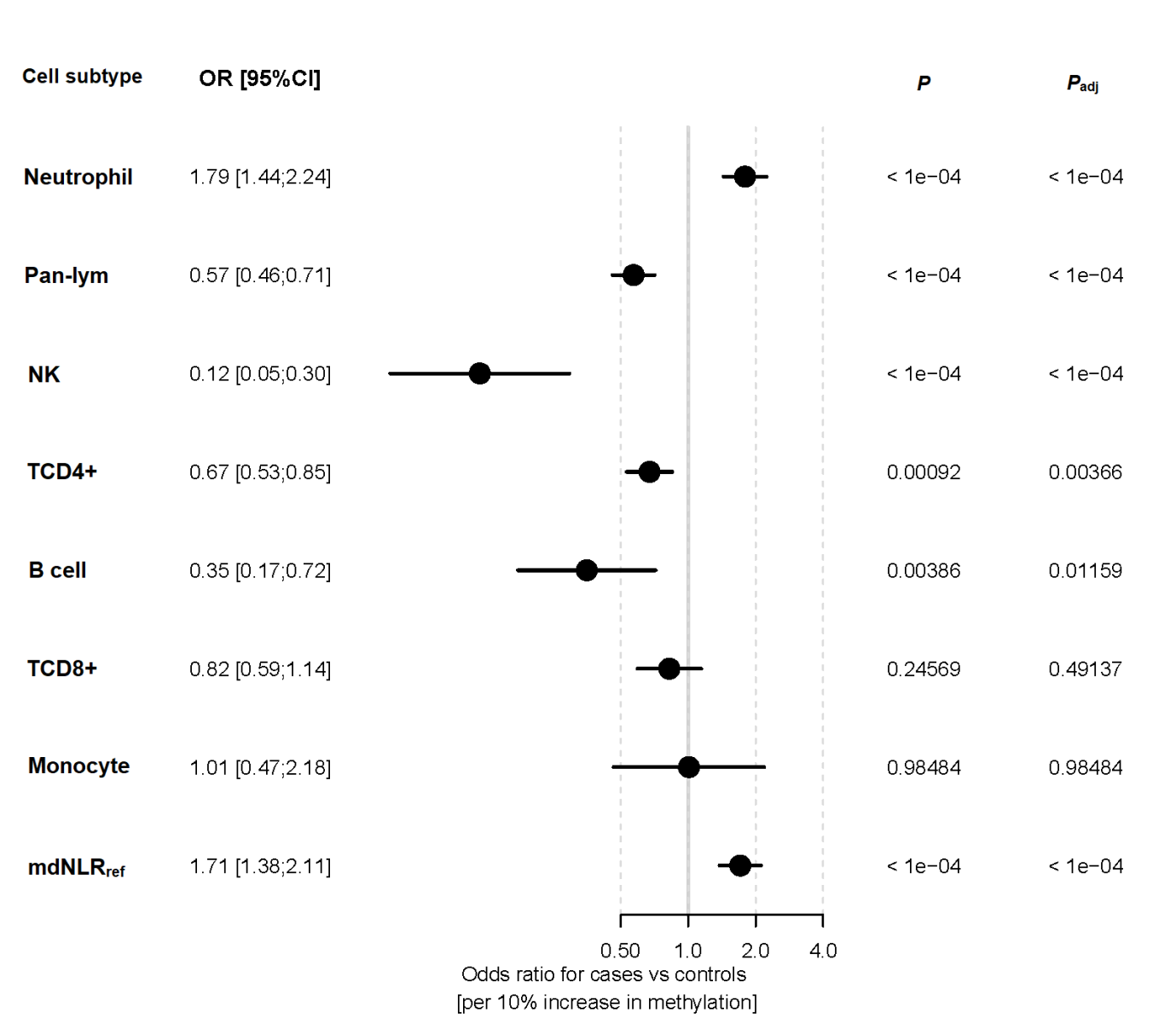


**Supplementary Figure 3:** Spearman's rank correlation scatter plot of mdNLR and mdNLR_ref_ levels in peripheral blood samples of TNBC cases and controls.

**
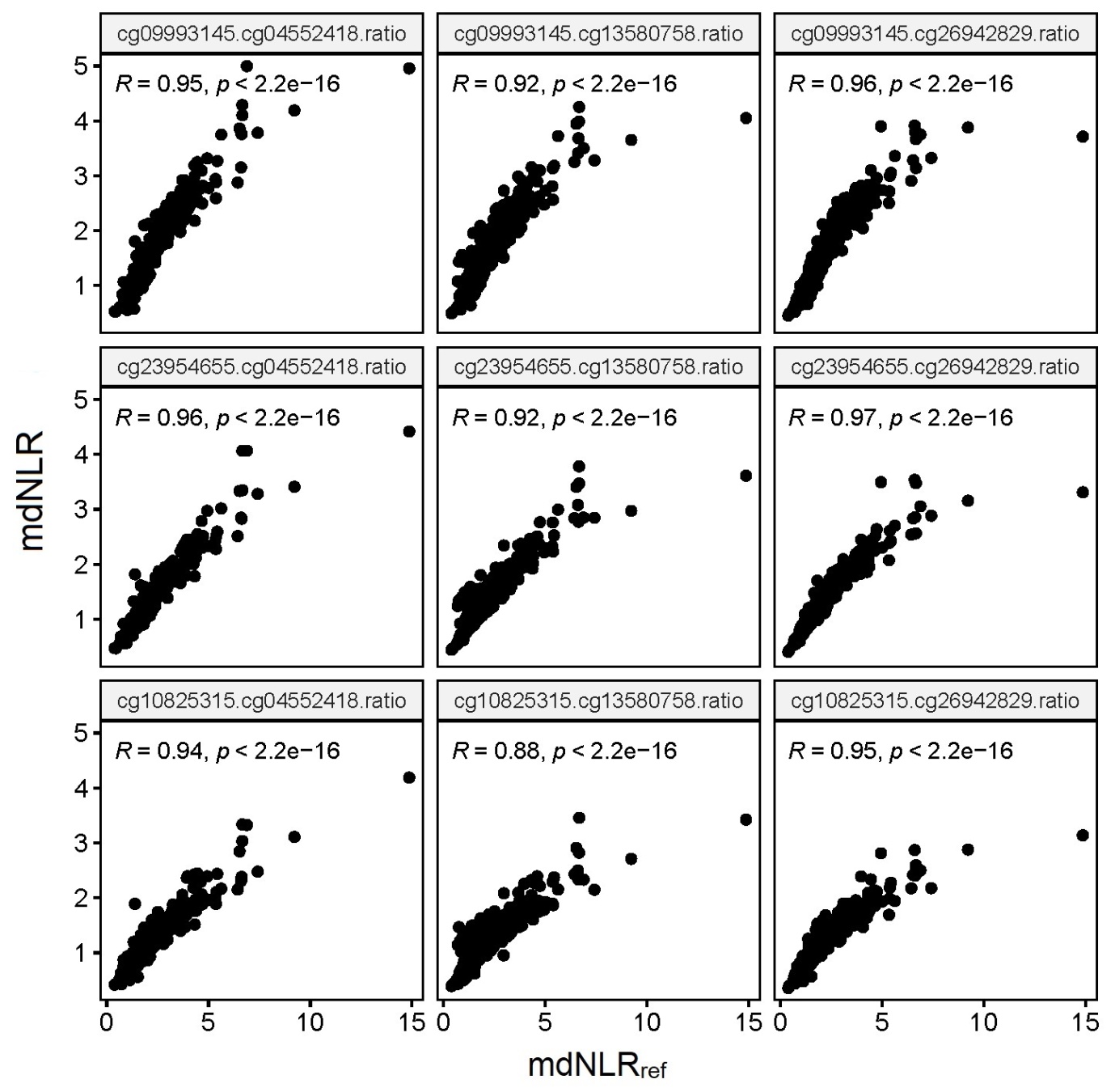
**

**Supplementary Figure 4.** Violin plots showing leukocyte subtype proportions in TNBC cases and controls estimated by Houseman method.

**
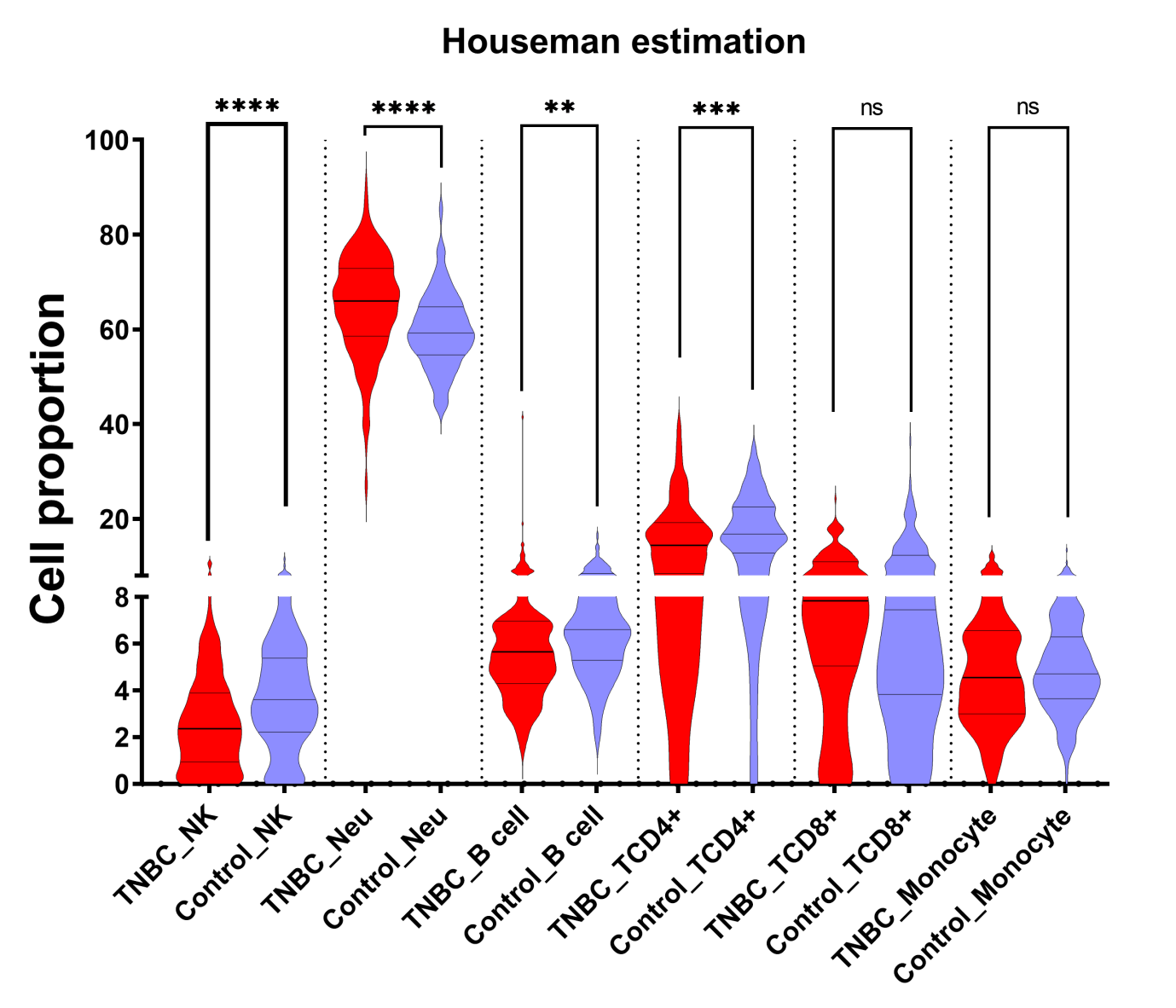
**

**Supplementary Figure. 5.** Area under the ROC curve of NK cell-to-neutrophil ratio in TNBC cases versus controls.


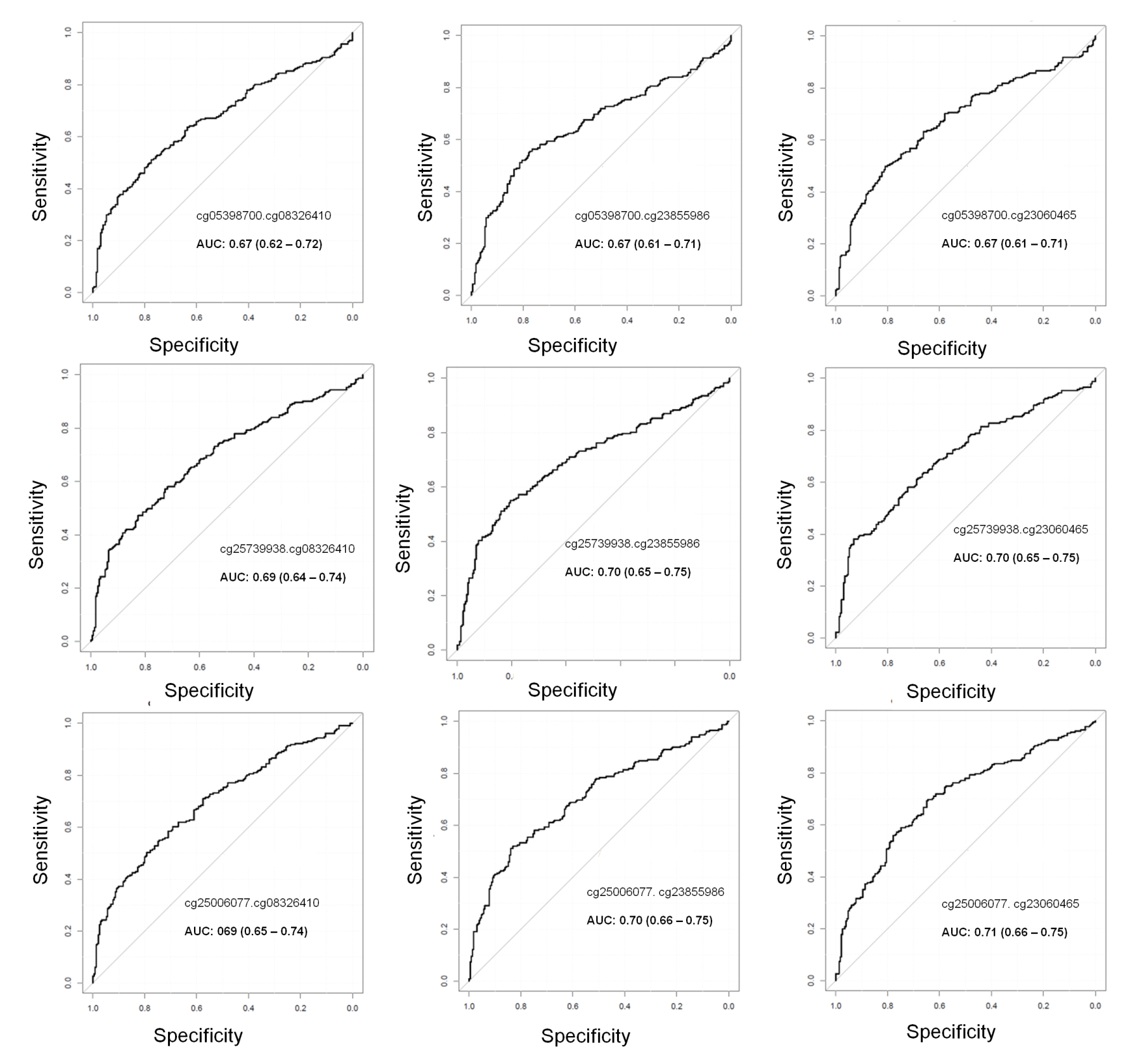


**Supplementary Figure 6.** Kaplan-Meier survival plots of cg08326419 and cg00219921 with prognostic value.


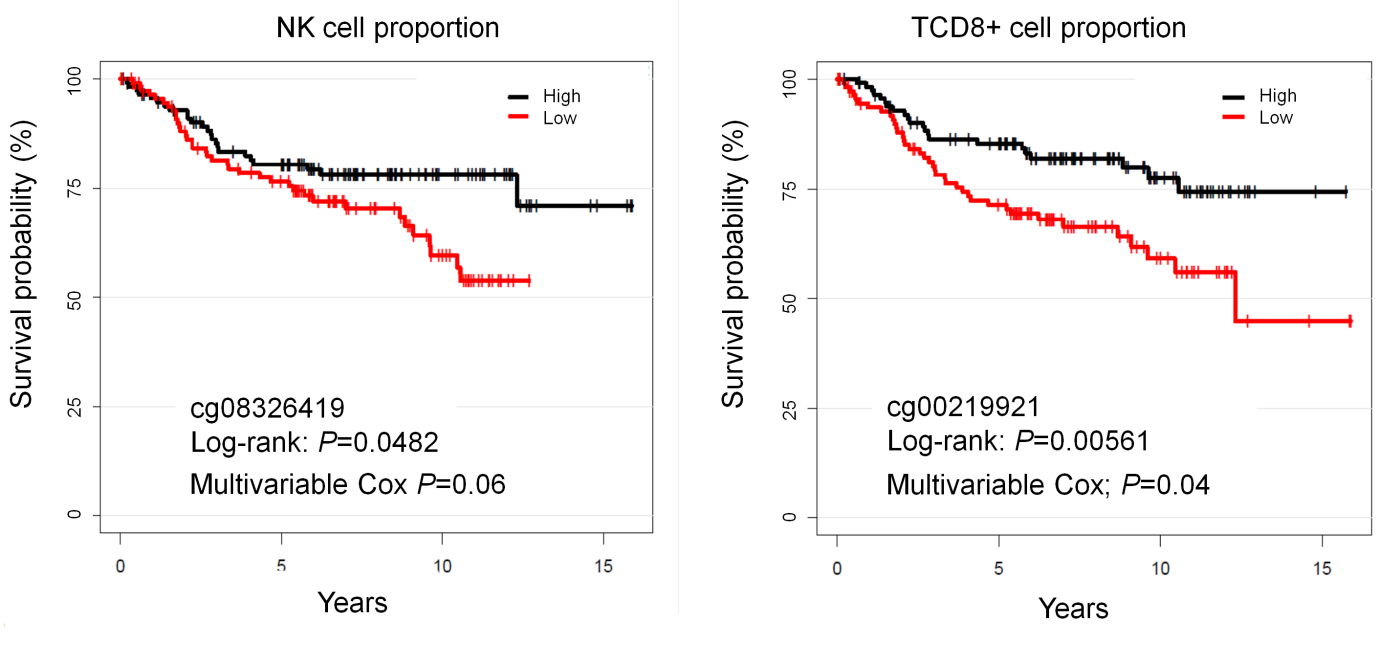

Supplement: Supplementary file 8 — Additional file 8: Fig. S1. Distribution of the mdNLRref between TNBC cases and controls using the reference-based Houseman method. Fig. S2. Forest plot of the associations of the immune cell subtype proportions and mdNLRref with TNBC. Odds ratio (OR) is given for a 10% increase in methylation level. P values were adjusted (Padj) for multiple testing using the Holm correction. Horizontal lines indicate 95% CIs.Fig. S3. Spearman's rank correlation scatter plot of mdNLR and mdNLRref levels in peripheral blood samples of TNBC cases and controls. Fig. S4. Violin plots showing leukocyte subtype proportions in TNBC cases and controls estimated by Houseman method. Fig. S5. Area under the ROC curve of NK cell-to-neutrophil ratio in TNBC cases versus controls. Fig. S6. Kaplan–Meier survival plots of cg08326419 and cg00219921 with prognostic value. [file 13148_2021_1196_MOESM8_ESM.docx]
