# Supplementary material for: Epigenetic quantification of circulating immune cells in peripheral blood of triple-negative breast cancer patients
Source: Clin Epigenetics. 2021 Nov 17;13:207. doi: 10.1186/s13148-021-01196-1 (PMC8596937; doi:10.1186/s13148-021-01196-1)
Supplement: Supplementary file 9 — Additional file 9. Supplementary methods [file 13148_2021_1196_MOESM9_ESM.docx]

**SUPPLEMENTARY MATERIALS**

**Supplementary Methods**

**Study populations**

**TNBC cases and controls from a retrospective study**

TNBC cases were selected from the Städtisches Klinikum Karlsruhe Deutsches Krebsforschungszentrum Breast Cancer Study (SKKDKFZS) and controls from the Gene Environment Interaction and Breast Cancer in Germany (GENICA) study. SKKDKFZS is a hospital-based breast cancer cohort study [[1](#_ENREF_1), [2](#_ENREF_2)]. It consists of 1,698 women diagnosed with *in situ* or invasive breast cancer recruited at the Städtisches Klinikum Karlsruhe from 03/1993 to 07/2005. Cases were between 21 and 93 years of age. Information on known and potential risk factors and follow-up was collected for all study participants from medical records, pathology reports and/or questionnaires. GENICA is a population-based breast cancer case-control study from the Greater Bonn Region [[3](#_ENREF_3), [4](#_ENREF_4)]. Cases and controls were recruited from 08/2000 to 09/2004. They were eligible when they were of Caucasian ethnicity, current residents of the study region, and below 80 years of age. The study consists of 1,021 women diagnosed with incident breast cancer and 1,015 controls matched in 5-year classes. Information of known and potential risk factors was collected for all participants via in-person interviews.

All TNBC cases met the following criteria: (1) negative for estrogen receptor, progesterone receptor (immunoreactive score <3 or lower ranking <20 fmol/mg protein), and epidermal growth factor 2 (scores 0, 1); (2) no neoadjuvant or adjuvant therapy before blood withdrawal; and (3) diagnosis before the age of 80 years. Controls were matched to cases by +/- one year of age. The median ages at diagnosis of TNBC in the retrospective case group was 58.3 years. The SKKDKFZS and GENICA study were approved by the ethics committees of the Medical Faculty of the University of Heidelberg and the University of Bonn, respectively.

**TNBC cases and controls from a prospective study**

The prospective subjects were from a nested case-control study in which participants were selected from the Generations Study (GS), a long-term prospective cohort study focused on potential etiological factors for breast cancer in women in the UK. In total, 146 TNBC cases diagnosed with a first triple-negative invasive or *in situ* primary breast cancer after study entry and 146 matched controls were included for the statistical analysis of methylation data. Controls were matched to cases based on age of blood draw, age at study entry +/- five-years, year of study entry, days blood in post before processing, and cancer-free years). The mean time from blood donation to the time of diagnosis in prospective cohort was 4.8 years. The study was approved, under the procedures for national medical research studies, by the South-East Multi-Centre Research Ethics Committee.

**DNA extraction, quantification and quality control**

Genomic DNA was extracted from 20 ml blood samples using proteinase K digestion and standard phenol–chloroform extraction. DNA samples were quantified using PicoGreen dsDNA quantification reagent (Invitrogen, Darmstadt, Germany). The quality of the DNA samples was checked by loading 100 ng of each sample on a 0.8% agarose gel stained with ethidium bromide. All DNA samples had fragment sizes greater than 3 kb and were suited for inclusion into the study.

**DNA bisulfite conversion**

After genomic DNA quantification, 1 μg of genomic DNA underwent bisulfite modification utilizing the EZ DNA Methylation-Direct Kit (Zymo Research, Orange, US). Thereafter, samples were purified using spin columns supplied with the EZ DNA Methylation Kit (Zymo Research). The DNA was suspended in 12 μl TE buffer, and stored at -80°C until methylation analysis.

**DNA methylation analysis**

Genome-wide DNA methylation profiling was performed on 233 TNBC cases and 233 controls from a retrospective study using the Illumina Infinium HumanMethylation450K BeadChip according to the manufacturer´s instructions. Data preprocessing was performed using Illumina GenomeStudio V2011.1 (Methylation Module version 1.9.0, content descriptor version 1.2). Two samples and their matched counterparts were excluded due to the poor array performance and outlying pattern in the principal component analysis. A detailed description of the DNA methylation analysis, data collection, quality control, and preprocessing was provided in our previous report [[5](#_ENREF_5)].

For measurement of the NK cell level in the prospective cohort of 146 TNBC cases and 147 controls, locus-specific DNA methylation analysis was carried out by the MethyLight droplet digital PCR (ddPCR) assay using region-specific primers and TaqMan probes as previously described [[5](#_ENREF_5)]. In brief, two hundred ng of DNA was extracted from blood were bisulfite-converted. The C-LESS-C1 assay was used as an internal control for normalization. Each reaction was performed in a final volume of 20 μl containing 10 μl ddPCR Supermix for Probes (No dUTP) (Bio-Rad, Hercules, CA, USA), 900 nM forward and reverse primers, 250 nM probe, 2 µl bisulfite converted DNA template, and 6 µl nuclease-free double distilled H_2_O. All ddPCR steps were performed according to the manufacturer’s protocols (Bio-Rad).

**Estimation of mdNLR using neutrophil- and pan-lymphocyte-specific methylation markers**

In order to estimate the mdNLR in the retrospective sample set of 231 TNBC cases and 231 controls, we first identified CpG sites specifically methylated in either neutrophils or pan-lymphocytes (NK + TCD4+ + TCD8+ + B cells). In this regard, we applied DNA methylation datasets from the Gene Expression Omnibus (GEO) obtained from various sorted leukocytes. One dataset (GSE35069) was used for discovery and two datasets (GSE88824 and GSE110554) for validation. Neutrophil-specific methylation markers were selected by differential methylation analysis of neutrophils against pan-lymphocytes and monocytes, and pan-lymphocyte-specific methylation markers by analysis of pan-lymphocytes against neutrophils and monocytes. The selected sites passed the following criteria: (i) The CpG site was methylated in the desired leukocytes compared with other leukocyte subtypes (Δβ ≥ 0.6), (ii) the selected site overlapped between 450K and EPIC arrays, (iii) and the site was validated in two other methylation datasets. Three neutrophil and three pan-lymphocytes-specific methylated sites were selected for the calculation of the mdNLR. The mdNLR was estimated by dividing the methylation ratio at neutrophil-specific CpG by the beta value of the pan-lymphocyte-specific CpG. The combination of the six CpG sites resulted in nine mdNLR proxies for each sample.

**Identification of immune cell-specific unmethylated sites**

In order to identify immune cell-specific unmethylated sites (ISUS) as proxies for immune cell types, we applied GEO DNA methylation datasets *o*btained from seven isolated leukocyte subtypes (NK, TCD4+, TCD8+, Treg cells, monocytes, neutrophils, and B cells). Two datasets (GSE35069 and GSE49667) were used for discovery and three datasets (GSE88824, GSE110554, and GSE59250) for validation [[6-10](#_ENREF_6)]. GEOquery and limma R packages were used to perform the comparisons using the GEO2R tool [[11](#_ENREF_11)]. The *P*-values were adjusted (*P_adj_*) for multiple testing using the Benjamini and Hochberg method [[1](#_ENREF_1)]. The selection of each ISUS was based on the following criteria: (i) The CpG was hypomethylated in the target cell type relative to the other cell types in the blood (Δβ ≥ 0.3) and was methylated in other leukocytes (mean β-value ≥ 0.85); (ii) the adjacent CpG probe (if available in <1000 bp distance) showed the same methylation direction and (iii) the selected site showed the same methylation profile in the validation datasets. For each of the seven leukocyte subtypes, three unmethylated CpG sites were selected.

**Immune cell estimation using the Houseman method**

For the retrospective cohort, we also estimated immune cell proportions using the Houseman algorithm [[12](#_ENREF_12)]. Data quality control, preprocessing and normalization using wm.dasen implemented in the wateRmelon package [[13](#_ENREF_13)] was done using RnBeads [[14](#_ENREF_14)]. Further data processing included removal of 24,948 cross-reactive probes [[15](#_ENREF_15)], 39,265 probes overlapping with SNPs (dbSNP Build 150, Feb. 2017) and 10,625 sites located on sex chromosomes. For cell type estimation, we used a reference blood cell sample set (GSE110554, [[9](#_ENREF_9)]) comprising neutrophils (n=6), monocytes (n=6), B cells (n=6), TCD4+ (n=6), TCD8+ (n=6), and NK (n=6) cells, hybridized to Illumina Infinium HumanMethylationEPIC BeadChips. We selected the 450 most discriminatory CpG sites between the cell types (rnb.options: inference.max.cell.type.markers = 50,000, inference.top.cell.type.markers = 450) from common probes (n=374,712) between both types of arrays. As control, we performed linear correlation analysis between ground truth and predicted cell type proportions of twelve DNA artificial mixtures (labeled as MIX) provided in the dataset (R2 = 0.9669).

**Supplementary References**

1. Stevens KN, Vachon CM, Lee AM*, et al.* Common breast cancer susceptibility loci are associated with triple-negative breast cancer. Cancer Res 2011;71(19):6240-9.

2. Stevens KN, Fredericksen Z, Vachon CM*, et al.* 19p13.1 is a triple-negative-specific breast cancer susceptibility locus. Cancer Res 2012;72(7):1795-803.

3. Pesch B, Ko Y, Brauch H*, et al.* Factors modifying the association between hormone-replacement therapy and breast cancer risk. Eur J Epidemiol 2005;20(8):699-711.

4. Justenhoven C, Pierl CB, Haas S*, et al.* The CYP1B1_1358_GG genotype is associated with estrogen receptor-negative breast cancer. Breast Cancer Res Treat 2008;111(1):171-7.

5. Bermejo JL, Huang G, Manoochehri M*, et al.* Long intergenic noncoding RNA 299 methylation in peripheral blood is a biomarker for triple-negative breast cancer. Epigenomics 2019;11(1):81-93.

6. Reinius LE, Acevedo N, Joerink M*, et al.* Differential DNA methylation in purified human blood cells: implications for cell lineage and studies on disease susceptibility. PLoS One 2012;7(7):e41361.

7. Zhang Y, Maksimovic J, Naselli G*, et al.* Genome-wide DNA methylation analysis identifies hypomethylated genes regulated by FOXP3 in human regulatory T cells. Blood 2013;122(16):2823-36.

8. Kennedy DW, White NM, Benton MC*, et al.* Critical evaluation of linear regression models for cell-subtype specific methylation signal from mixed blood cell DNA. PLoS One 2018;13(12):e0208915.

9. Salas LA, Koestler DC, Butler RA*, et al.* An optimized library for reference-based deconvolution of whole-blood biospecimens assayed using the Illumina HumanMethylationEPIC BeadArray. Genome Biol 2018;19(1):64.

10. Absher DM, Li X, Waite LL*, et al.* Genome-wide DNA methylation analysis of systemic lupus erythematosus reveals persistent hypomethylation of interferon genes and compositional changes to CD4+ T-cell populations. PLoS Genet 2013;9(8):e1003678.

11. Davis S, Meltzer PS. GEOquery: a bridge between the Gene Expression Omnibus (GEO) and BioConductor. Bioinformatics 2007;23(14):1846-7.

12. Houseman EA, Accomando WP, Koestler DC*, et al.* DNA methylation arrays as surrogate measures of cell mixture distribution. BMC Bioinformatics 2012;13:86.

13. Pidsley R, CC YW, Volta M*, et al.* A data-driven approach to preprocessing Illumina 450K methylation array data. BMC Genomics 2013;14:293.

14. Muller F, Scherer M, Assenov Y*, et al.* RnBeads 2.0: comprehensive analysis of DNA methylation data. Genome Biol 2019;20(1):55.

15. Chen YA, Lemire M, Choufani S*, et al.* Discovery of cross-reactive probes and polymorphic CpGs in the Illumina Infinium HumanMethylation450 microarray. Epigenetics 2013;8(2):203-9.
